# The Lumenal Microbiota Varies Biogeographically in the Gastrointestinal Tract of Rhesus Macaques

**DOI:** 10.1128/spectrum.00343-22

**Published:** 2022-05-02

**Authors:** Yaping Yan, Junfeng Wang, Shuai Qiu, Yanchao Duan, Wei Si

**Affiliations:** a State Key Laboratory of Primate Biomedical Research, Institute of Primate Translational Medicine, Kunming University of Science and Technology, Kunming, Yunnan, China; b Digital Medical Research Center, Department of Hepatobiliary Surgery, the First People’s Hospital of Yunnan Province, Kunhua Hospital Affiliated to Kunming University of Science and Technology, Kunming, China; Lerner Research Institute

**Keywords:** rhesus monkey, gastrointestinal tract, biogeographically, microbiota diversity, composition, gut microbiota

## Abstract

The strategy of adjusting the composition of gastrointestinal microbiota has shown great promise for the treatment of diseases. Currently, the relationship between gut microbes and human diseases is mainly presented by the fecal microbiota from the colon. Due to the limits of sampling, the healthy baseline of biogeographic microbiota in the human gastrointestinal tract remains blurry. Captive nonhuman primates (NHPs) present a “humanized” intestinal microbiome and may make up for the lack of atlas data for better understanding of the gut microbial composition and diseases. Therefore, the intestinal microbiota of 6 GIT regions of healthy rhesus monkeys were analyzed in this study; our results showed that *Proteobacteria* gradually decreased from the small intestine to the large intestine but *Bacteroidetes* gradually increased from the small intestine to the large intestine. Streptococcus and *Lactobacillus* can be used as markers to distinguish the small intestine from the large intestine. *Sarcina* is the most enriched in the middle site of the connection between the large intestine and the small intestine. *Cyanobacteria* are enriched in the small intestine, especially the duodenum and jejunum, and are absent in the large intestine. The lumenal microbiota of the small intestine is more susceptible to individual differences than is that of the large intestine. Metabolism and oxygen affect the distribution of the microbes, and the diversity of microbiota is the highest in the colon. Our results provide accurate comprehensive GIT microbiota data on nonhuman primates and will be beneficial for the better understanding of the composition of microbiota in the human gastrointestinal tract.

**IMPORTANCE** For the study of upper gastrointestinal microbiota in humans, endoscopic sampling is the main source of information, which limits the understanding of healthy upper gastrointestinal microbiota. Rhesus monkeys show very close similarity to humans in physiology, genetics, and behavior and act as the most suitable animal models for human diseases. The present research made up for the lack of atlas data due to the ethical limitations of sampling in humans and provided baseline data on microbiota in 6 GIT regions of healthy NHPs. These important references will be beneficial for the better understanding of the regional organization and functions of gut microbial communities along the GIT and their relevance to conditions of health and disease.

## INTRODUCTION

The gastrointestinal tract (GIT) is a multiorgan system with great regional diversity, which contains a wide range of gut microbes and provides diverse functions. Each region of the GIT is geographically specialized in gene expression and function. The small intestine (SI) secretes pancreatic juice and bile acids (BAs) to break down nutrients into absorbable small molecules that can enter the blood or lymph circulation (1). The large intestine (LI) further absorbs water in food, and the mucus secreted by the LI can protect the intestinal wall from mechanical damage and prevent bacterial erosion (2). Oxygen levels, nutrient utilization, pH, bile acids, digestion time, mucus, and immune factors in different regions are all important determinants for the selection of symbiotic microbiota (3). The acquisition of the regional microbial community is not random but comes from actions to meet specific host needs through dynamic mutual reinforcement and mutual assistance, which regulate the complex and diverse digestion, immunity, metabolism, and endocrine processes (4, 5).

Due to sampling limits, current research on the relationship between gut microbes and human diseases mainly focuses on the fecal microbiota from the colon. The colon has the densest and most diverse communities of microbiota (6). The entire wall of the colon is folded, and the folded area contains abundant mucus, which can be used as a source of nutrients for certain bacteria in addition to digestion (7). Meanwhile, the sampling of colon contents is convenient for the study of microbiota and diseases. Various host factors drive community differences on the intestinal cross-sectional axis. It may not be universally applicable or appropriate to study various diseases presented by the microbes only in the colon. After all, each region of the intestine has unique functions and microbial compositions. It has been proven that some diseases can be potentially treated by adjusting the composition of the gut microbes. For example, fecal microbiota transplantation (FMT) has been applied in clinical treatment of gastrointestinal diseases (8–10), but the distribution of the microbes in various regions of the GIT is not completely clear, which also brings safety concerns to the clinical application of FMT (11). The lumenal contents of mice are widely used in biogeographical microbiota research. However, neither the granular, sparse nature of their colon contents nor their natural microbial composition can fully represent the human intestine (12). Previous studies on the microbiota of human intestinal biogeography involved only a limited number of individuals and GIT regions (13–15). Furthermore, the individuals involved usually were patients with gastrointestinal diseases, who scarcely could present the normal microbiota communities of human GIT regions. Previous studies have reported the microbial composition of 10 locations in the gastrointestinal tract of 15 macaques, including the intestinal lumen and intestinal mucosa. Intestinal lumen samples (ileum and ascending, transverse, and distal colon), mucosal scrapings (jejunum, ileum, and ascending, transverse, and descending colon), and stool samples were collected. The results of this study found that obligate anaerobic *Firmicutes* were primarily localized to the intestinal lumen. Small intestinal proteobacteria are particularly undetected in stool. However, in this study jejunal lumenal contents were not collected due to difficulties in sampling, and ileal lumenal contents were obtained from 4 rhesus monkeys. In addition to the jejunum, the lumenal microbes of the duodenum and cecum were also not analyzed (16). Therefore, the biogeographic GIT microbiota of a healthy baseline remains incomplete.

Studies investigating the differences in the gut microbiome between captive and wild nonhuman primates (NHPs) found that the captive NHPs present a “humanized” intestinal microbiome (16, 17). Similar to the human microbiome, the captive rhesus macaques are enriched with *Bacteroidetes*, *Firmicutes*, and *Proteobacteria* in fecal matter (16, 18). In addition, the captive rhesus macaque has served as the ideal animal model in biomedical research due to its similarity to humans in physiology, metabolism, genetics. and behavior. Therefore, the NHP disease models play essential roles in human disease research and drug development, which also offer great promise for therapeutic strategies to be validated in research on microbial drugs (19). Since the knowledge on biogeographic GIT microbiota in humans is not comprehensive, the present study is intended to explore the composition and diversity of the microbes in the various GIT regions of rhesus monkeys including the duodenum (DU), jejunum (JE), ileum (IL), cecum (CE), colon (CO), and rectum (RE) and to provide accurate comprehensive microbiota data for the study of human microbes and diseases.

## RESULTS

### Lumenal microbial diversity from six GIT regions of rhesus monkeys.

**FIG 1:**
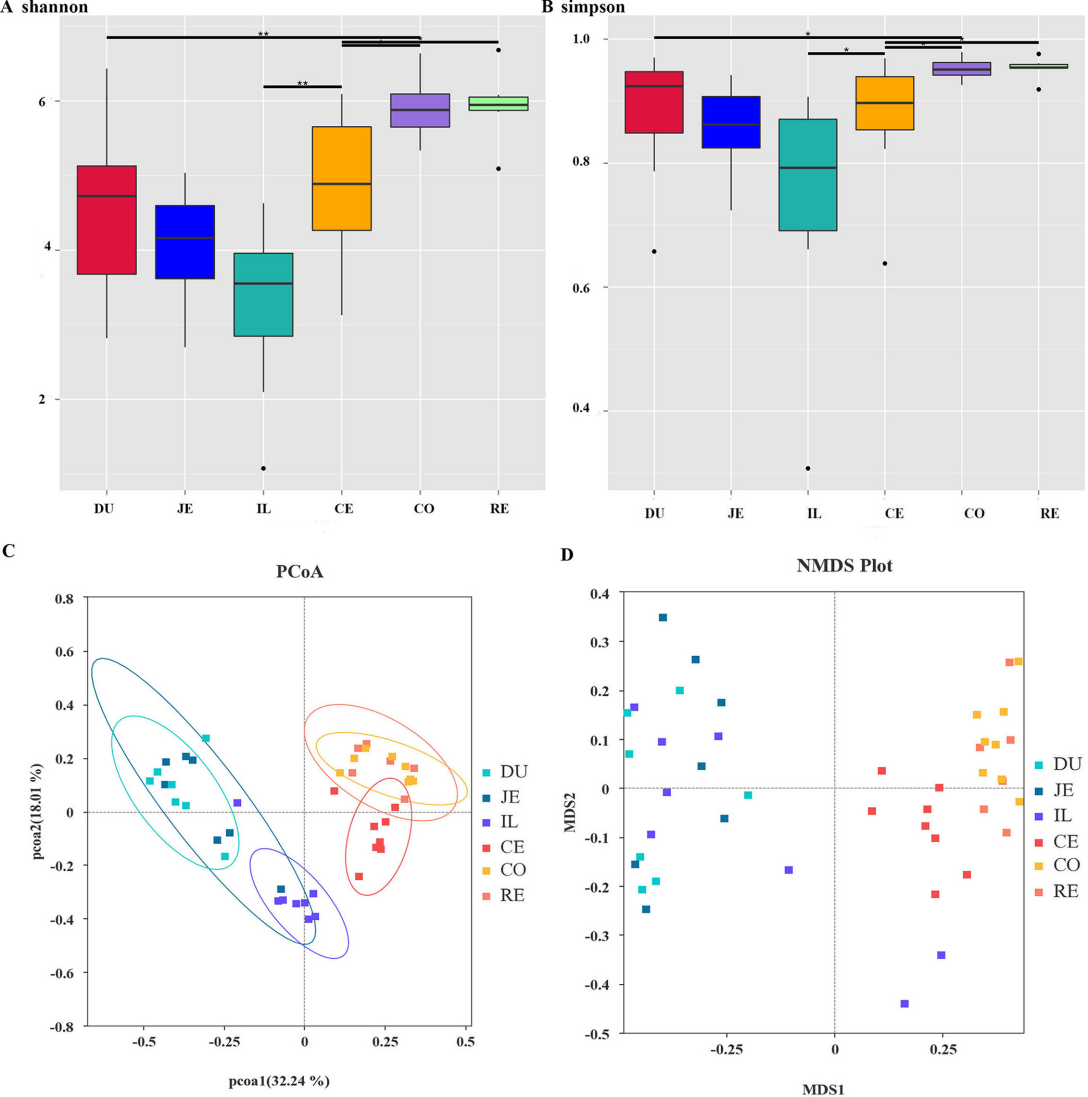
Gastrointestinal biogeography influences gut microbial diversity in macaques, including the Shannon index (A) and Simpson index (B). Beta diversity analysis includes PCoA (C) and nonmetric multidimensional scaling (NMDS) (D).

Alpha diversity is used to analyze the diversity of microbial communities in samples (within the community) including the Shannon and Simpson indices. The Wilcox rank sum test was used to analyze species diversity among groups. The Shannon index and Simpson index indicate that significant differences in species diversity exist between the duodenum and the colon, the ileum and the cecum, the cecum and the colon, and the cecum and the rectum, respectively (Fig. 1A and B). The highest species diversity was observed in the colon.

Beta diversity presents a comparative analysis of the composition of microbial communities in different samples. Principal-coordinate analysis (PCoA) was performed based on unweighted UniFrac distance, and nonmetric multidimensional scaling (NMDS) analysis was conducted based on Bray-Curtis distance. The microbiota composition showed an obvious separation in the small intestine and large intestine by PCoA and NMDS analysis (Fig. 1C and D).

### Compositions of lumenal microbiota from six GIT regions of rhesus monkeys.

**FIG 2:**
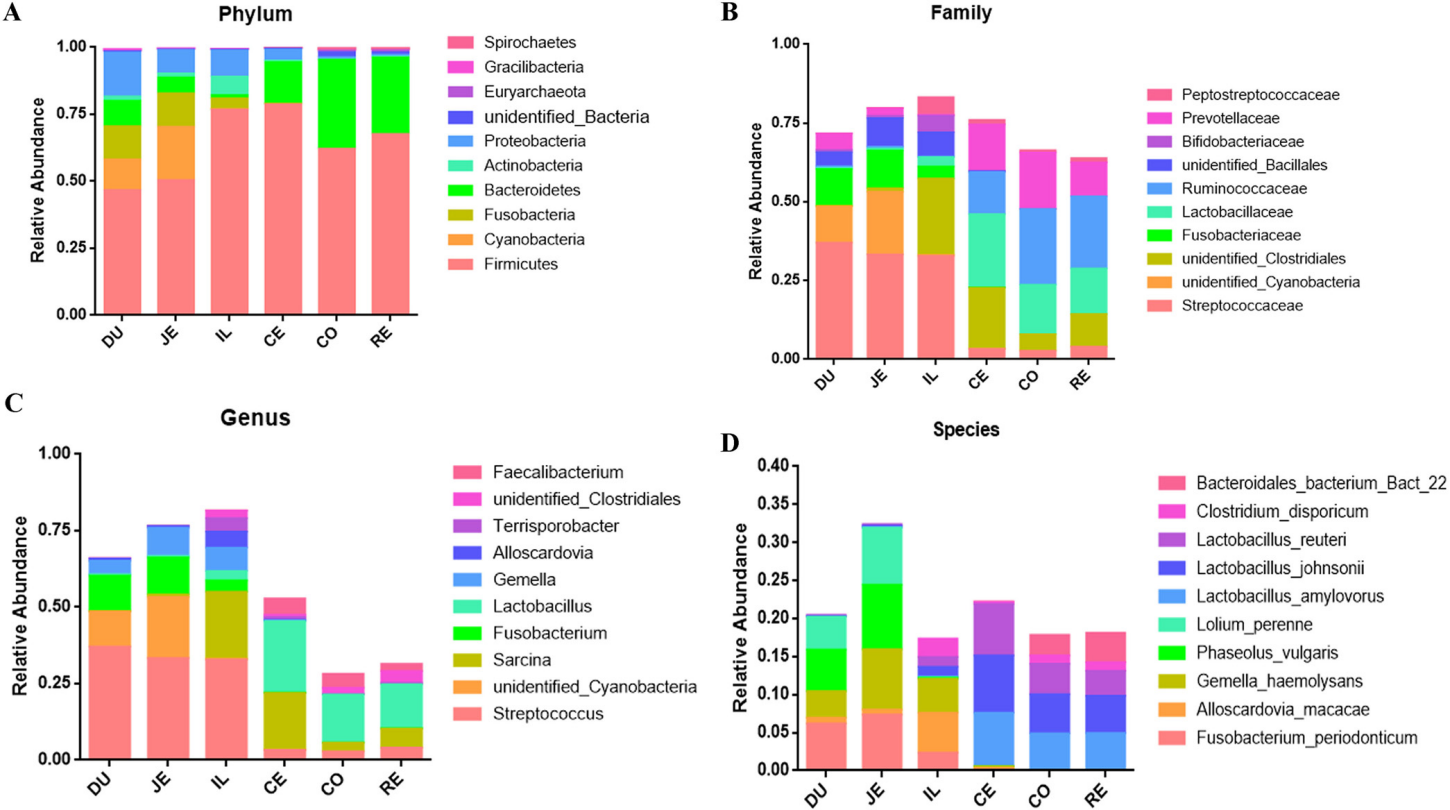
Composition of microbiota from six GIT regions. (A) Composition of microbiota at phylum level. (B) Composition of microbiota at family level. (C) Composition of microbiota at genus level. (D) Composition of microbiota at species level.

The relative abundance of *Bacteroidetes* gradually increased from the duodenum to the rectum. The relative abundance of *Fusobacteria* and *Proteobacteria* gradually decreased from the duodenum to the rectum (Fig. 2A). The relative abundance of *Streptococcaceae* and *Fusobacteriaceae* decreased gradually from the duodenum to the rectum. The relative abundance of *Ruminococcaceae* increased from the duodenum to the rectum (Fig. 2B). The relative abundance of *Lactobacillus* is higher in the cecum, colon, and rectum. *Lactobacillus* was not found in the duodenum and jejunum. *Gemella* is enriched only in the duodenum, jejunum, and ileum. *Sarcina* is enriched mainly in the ileum and cecum (Fig. 2C). Phaseolus vulgaris and Lolium perenne from the plants were enriched in the duodenum and jejunum. Lactobacillus amylovorus, Lactobacillus johnsonii, and Lactobacillus reuteri are enriched mainly in the cecum, colon, and rectum (Fig. 2D). The differences in the microbe abundances at three taxonomic levels (phylum, family, and genus) among the 6 GIT regions are summarized in Table 1.

**TABLE 1:** Difference of the abundances of microbiota at three taxonomic levels (phylum, family, and genus) among the 6 six GIT regions

Region	Phyla	Families	Genera
Duodenum	*Gracilibacteria*, *Proteobacteria*, *Fusobacteria*	*Porphyromonadaceae*, *Streptococcaceae*, *Pasteurellaceae*, *Neisseriaceae*	Streptococcus, *Gemella*, *Porphyromonas*, *Fusobacterium*, *Veillonella*, *Granulicatella*
Jejunum	*Cyanobacteria*	*Fusobacteriaceae*	Streptococcus, *Sarcina*, *Gemella*, *Porphyromonas*, *Fusobacterium*, *Veillonella*, *Granulicatella*
Ileum	*Actinobacteria*, *Proteobacteria*	*Bifidobacteriaceae*, *Peptostreptococcaceae*	Streptococcus, *Sarcina*, *Gemella*, *Porphyromonas*, *Fusobacterium*, *Veillonella*, *Granulicatella*
Cecum	*Firmicutes*, *Spirochaetes*	*Lachnospiraceae*, *Erysipelotrichaceae*	Streptococcus, *Sarcina*, *Lactobacillus*, *Faecalibacterium*
Colon	*Bacteroidetes*, *Spirochaetes*	*Prevotellaceae*, *Ruminococcaceae*	*Sarcina*, *Lactobacillus*, *Faecalibacterium*
Rectum	*Euryarchaeota*, *Spirochaetes*	*Muribaculaceae*, *Rikenellaceae*, *Christensenellaceae*	Streptococcus, *Sarcina*, *Lactobacillus*, *Faecalibacterium*

### Typical microbiota at six geographic locations in intestines of rhesus monkeys.

**FIG 3:**
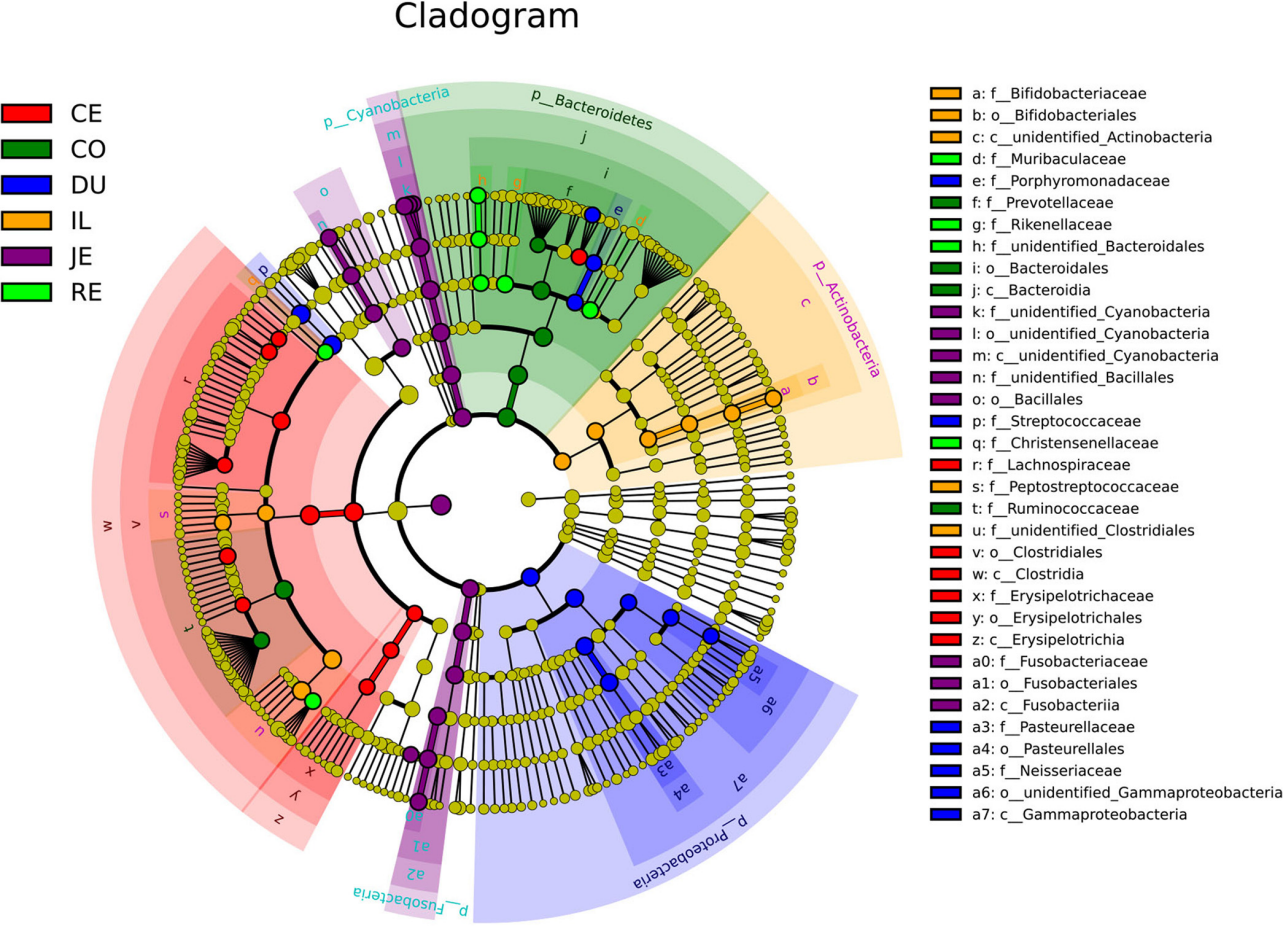
The cladogram by the LEfSe method indicates the phylogenetic distribution of gut microbiota associated with the six GIT regions.

We analyzed the typical microbiota in each area by the linear discriminant analysis effect size (LEfSe) method. *Proteobacteria* are typical microbiota in the duodenum. *Fusobacteria* are typical microbiota in the jejunum. *Actinobacteria* and *Bifidobacteriaceae* are representative microbiota in the ileum. *Lachnospiraceae* and *Clostridia* are characteristic microbiota in the cecum. *Prevotellaceae* and *Ruminococcaceae* are representative microbiota in the colon. *Rikenellaceae* and *Christensenellaceae* are characteristic microbiota in the rectum (Fig. 3).

### Alteration of taxa between the small intestine and large intestine of rhesus monkeys.

**FIG 4:**
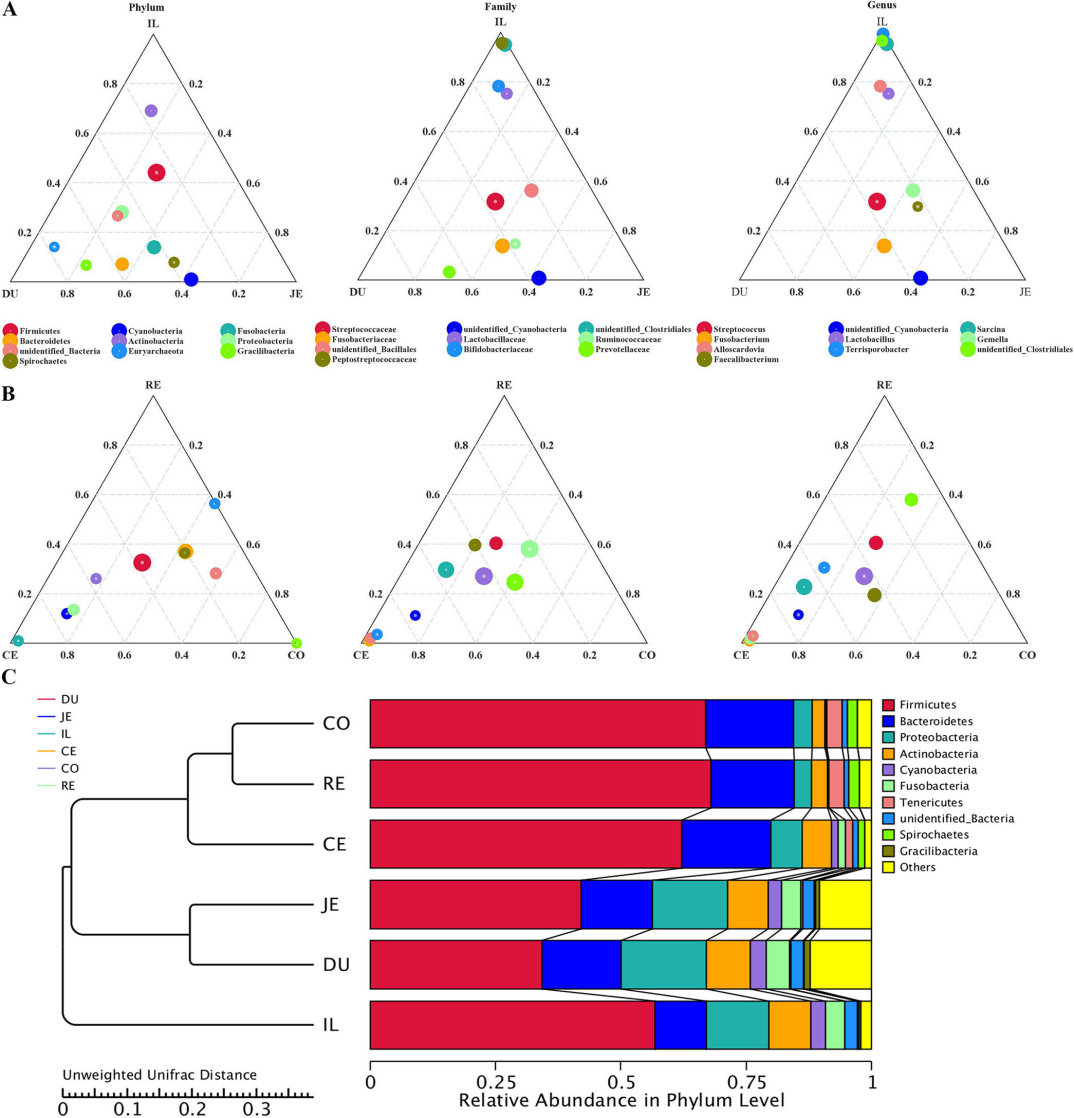
Comparation of the intestinal microbial compositions between the large intestine and the small intestine. (A and B) The three vertices represent phylum, family, and genus, and the circles represent the different microbiota at the three levels. The size of the circle is proportional to the relative abundance; the closer the circle is to the vertex, the higher the content of this species in this group. (C) Cluster tree analysis in the six GIT regions. On the left is the UPGMA cluster tree structure; on the right is the relative abundance distribution of species at the gate level of each sample.

In order to identify the differences in microbiota between LI and SI of rhesus monkeys, we performed a ternary plot analysis. The results showed that the relative abundance of *Firmicutes* was highest at 6 locations. *Proteobacteria* (*P* = 0.001), *Cyanobacteria* (*P* = 0.051), and *Fusobacteria* (*P* = 0.016) are the most abundant phyla in the SI, and *Bacteroidetes* (*P* < 0.001) is the most abundant phylum in the LI. At the family level, *Streptococcaceae* (*P* < 0.001) and *Fusobacteriaceae* (*P* < 0.001) are the most abundant in the SI. *Ruminococcaceae* (*P* < 0.001), *Lactobacillaceae* (*P* = 0.001), and *Prevotellaceae* (*P* < 0.001) are the most abundant in the LI. At the genus level, Streptococcus (*P* < 0.001) and *Fusobacterium* (*P* < 0.001) are the most abundant in the SI. *Lactobacillus* (*P* = 0.002) and *Faecalibacterium* (*P* < 0.001) are the most abundant in the LI (Fig. 4A and B). We performed cluster analysis on the six GIT regions and constructed a cluster tree. The diversities of the microbiota are very similar in the duodenum and jejunum. Similarly, the difference of microbial diversity between the colon and the rectum is negligible. The composition of microbiota from the ileum is independent of the other five regions (Fig. 4C). We found that the colon and rectum share 90% of the operational taxonomic units (OTUs). At the three levels of phylum, family, and genus, the microbial compositions of the colon and rectum lumen are highly similar. These data suggest that stool microbial composition accurately reflects the colonic lumen.

### Predictive function analysis of six GIT regions.

**FIG 5:**
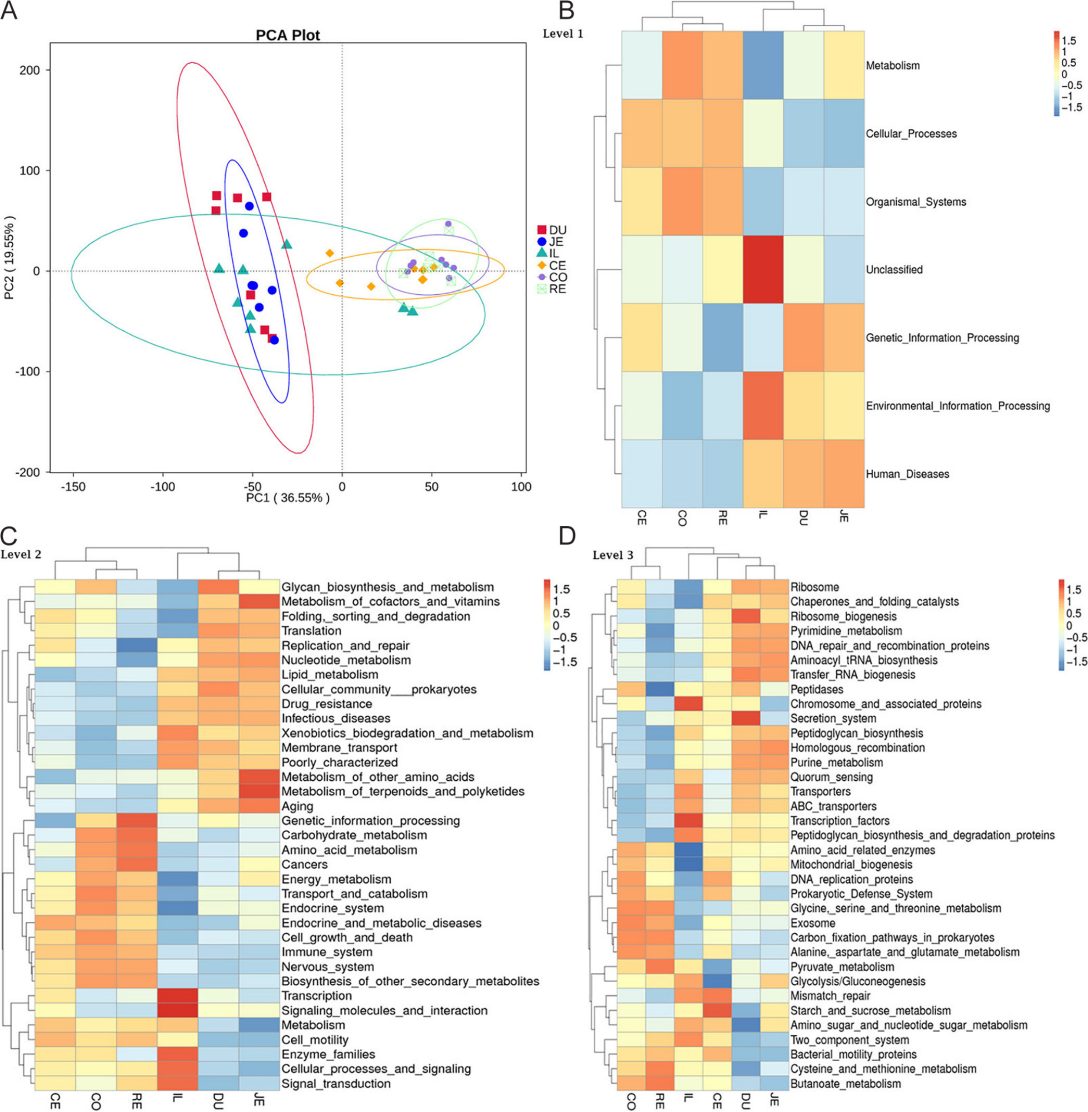
Function prediction analysis of intestinal microbiota in six GIT regions. (A) PCA based on the abundance statistical results of function annotations. (B to D) The function of the microbiota in the six GIT regions is classified hierarchically according to the composition of microbiota.

We used Tax4Fun for the function prediction analysis. The principal-component analysis (PCA) of the LI and SI is shown in Fig. 5A. Similar to the results for the composition of microbiota, the functional annotation of microbiota in the ileum is scattered. At the same time, the functional annotation of microbiota in the cecum is also relatively independent compared to the colon and rectum. Carbohydrate metabolism, amino acid metabolism, and energy metabolism were correspondingly increased in the colon and rectum. Glycolysis capacity was increased in the ileum (Fig. 5B to D).

## DISCUSSION

In this study, we analyzed the composition of intestinal microbiota in six GIT regions of 8 healthy individual macaques. The current study also found that age affects the composition of the gut microbiota (20, 21). In our study, the 8 macaques (7 males and 1 female) were 13 to 16 years old. These monkeys were kept in a uniform environment on the same diet for at least 2 years before sampling. Therefore, the influence of diet, drugs, and environment on this study is excluded, and the results should be more objective. The alpha diversity analysis showed that the diversity of microbiota in the colon is the highest, followed by that in the rectum. In contrast, the microbiota in the ileum shows the lowest diversity. The density and diversity of bacteria in the large intestine reach saturation. Bacteria in the small intestine may compete with the host for nutrition, but host-derived bile acids and antimicrobial peptides limit bacterial growth (22).

The diversity and composition of gut microbiomes are fundamentally similar in *Bacteroidetes*, *Firmicutes*, and *Proteobacteria* among humans, NHPs, and mice (23, 24). Our results show that the richness of the three phyla is not consistent in all of the GIT regions of monkeys. In our study, the richness of *Bacteroidetes* and *Firmicutes* gradually increased from the proximal to distal regions of the intestine and reached the highest levels in the colon and cecum, respectively. The relative abundance of *Firmicutes* was highest at 6 locations. *Proteobacteria* and *Fusobacteria* are mainly detected in the SI; these results were similar to those reported by Yasuda et al. (16). That previous study reported that the mucosal microbiota of macaques varied most based on location, while the lumenal microbiota showed interindividual variation. The 15 macaques (12 to 22 years old) varied widely in age and lived in two different U.S. states in this study. Due to sampling limitations, the sampling sites in the upper gastrointestinal tract and the number of animals involved in sampling were small (16). Comparison of the intestinal flora of Italian centenarians and centenarians from Sichuan, China, also found that only 11 genera were shared (25). Furthermore, obvious differences also were found between young and elderly subjects (26). So, diet, age, and living environment affect the composition of the intestinal microbiota (26–29). Our study also found that the lumenal microbiota composition of the upper gastrointestinal tract is greatly influenced by individuals. Previous studies of gut microbiota for rhesus macaques found that stool composition correlated highly with the colonic lumen and mucosa (16). We further compared the gut microbiota from colonic and rectal lumenal contents in the present study to the fecal microbiota of normal rhesus macaques reported in our previous study (30), in which the rhesus monkeys were fed in the same environment and at similar ages (10 to 15 years old) and under healthy conditions. We found that the compositions of gut microbiota are very similar, mainly including *Euryarchaeota*, *Spirochaetes*, Streptococcus, *Sarcina*, and *Lactobacillus*. Therefore, these data also suggest that the composition of the fecal microbiome is highly similar to that of the lumen of the colon and rectum. In humans, *Proteobacteria* are mainly enriched in the small intestine (31). In our results, we clarified that the abundance of *Proteobacteria* is region dependent in the small intestine and shows the most richness in the duodenum. In addition to *Proteobacteria*, *Cyanobacteria* and *Fusobacteria* also are abundant in the duodenum and jejunum of monkeys in our study. Studies have shown that *Fusobacteria* are enriched in the esophagi of humans (32). These combined data showed that *Fusobacteria* are mainly displayed in the upper digestive tract in humans and NHPs. *Bacteroidetes*, *Ruminococcaceae*, and *Lactobacillus* were enriched in the colon of monkeys, and this result is consistent with the microbial composition of the human colon (33). However, *Fusobacteria* and *Gracilibacteria* were mainly detected in the duodenal lumen of macaques. The relative abundances of *Cyanobacteria* and *Fusobacteriaceae* were highest in the jejunum, but *Fusobacteria*, *Gracilibacteria*, *Cyanobacteria* and *Fusobacteriaceae* were not found in the human duodenum and jejunum (34, 35). The reason may be that endoscopic sampling is the primary source of information on the composition of microbiota in the human upper gastrointestinal tract, which may not be representative of a healthy upper gastrointestinal microbiome (35). Also, diet, gastrointestinal motility, and medication history have the greatest impact on the gut microbiome (26–29). The oxygen content in the intestine seems to determine the dominant taxa in colonization of the SI and LI. This in turn dictates the main microbe patterns of region functionality, such as *Prevotellaceae*, *Lachnospiraceae*, and *Ruminococcaceae* being as a result primarily carbohydrate fermenters (36). These data make up for missing data on microbes in the duodenum, jejunum, ileum, and cecum in healthy humans without gastrointestinal diseases.

Our functional prediction analysis shows that glycan, lipid, amino acid, and vitamin metabolism was more active in the duodenum and jejunum. In the distal ileum, there are nuclear receptors in bile acid (BA) signal transduction, such as farnesoid X receptor (FXR) and G protein-coupled receptor TGR5 (37). Signal molecule interaction and signal transduction were the main functions of microbiota in the ileum in our study. The cecum and colon are the main regions of the conversion of primary BAs into more than 20 secondary BAs through an extensive microbial process (37). The peristaltic velocity of the GIT is related to the production of 5-hydroxytryptamine by the microbiota in the colon, so there is a bidirectional communication between the host and the microbiota in the colon (38, 39). Our results also show that the metabolism of secondary metabolites, energy metabolism, cell growth, and death are the main functions of microbiota in the colon. Functional annotation analysis showed that the microbial functions of the six regions of nonhuman primates are different, which may indicate that it is necessary to select the microbes of the corresponding region according to the pathogenic mechanism of the disease when studying diseases and intestinal microbes.

For the study of upper gastrointestinal microbiota in humans, endoscopic sampling is the main source of information, which limits the understanding of healthy upper gastrointestinal microbiota, because people who undergo endoscopic sampling usually suffer from some potential diseases such as colitis, rectal cancer, and so on (40, 41). Furthermore, endoscopic sampling alters the microbiota community. Although the contents of the digestive tracts of mice are easy for biogeographic research of the GIT, the granular, sparse nature and native microbial composition of the contents of the colon do not fully represent the human intestine (24). Nonhuman primates (NHPs) are the most biologically meaningful animal models for studying humans because they are our closest biological relatives. Despite differences in the gut microbiota between humans and nonhuman primates, exploring the gut microbiota composition of nonhuman primates can provide a better understanding of the regional organization and functions of gut microbial communities along the GIT and their relevance to conditions of health and disease.

## MATERIALS AND METHODS

### Animals.

Eight healthy rhesus monkeys (12 to 16 years old) were involved in this study (see Table S1 in the supplemental material). All of the animals were maintained in a 12-h light–12-h darkness cycle. The temperature and humidity of the animal rooms were kept at 18 to 26°C and 40% to 70%, respectively. The animals were fed twice per day with monkey chow. Fresh fruits and vegetables (including apples, bananas, pears, onions, and cabbages) were provided as supplements once per day. The lumenal contents from the duodenum (DU), jejunum (JE), ileum (IL), cecum (CE), colon (CO), and rectum (RE) were collected from the 8 monkeys after euthanasia. All procedures were approved by the Institutional Animal Care and Use Committee of Kunming University of Science and Technology (IACUC: LPBR201803006) and were carried out in accordance with the *Guide for the Care and Use of Laboratory Animals* (42).

### Method of dividing the 6 regions of the GIT.

The small bowel is divided into three sections. The first section is the duodenum, which is behind the pylorus. The duodenum from the jejunum is marked by the ligament of Treitz. There is no obvious difference between the jejunum and the ileum. However, the character of the small intestine does change as it is followed distally toward the cecum. The cecum is thick and short, which makes it easy to distinguish. The rectum connects to the anus, and between the cecum and rectum is the colon. The sampling of each part is selected at the middle point of the area, and the sampling position of 8 monkeys is guaranteed to be the same.

### Sample collection and DNA extraction.

The animals were euthanized by intravenous injection of 100 mg of sodium pentobarbital per kg of body weight. Then, the samples were transferred to the laboratory immediately in an ice bath and stored at −80°C. Total genome DNA from samples was extracted using the cetyltrimethylammonium bromide (CTAB)-SDS method. DNA concentration and purity were monitored on 1% agarose gels. According to the concentration, DNA was diluted to 1 ng/μL using sterile water.

### Amplicon generation.

The 16S rRNA genes of distinct regions (16S V3-V4) were amplified using specific primers (16S V3-V4, 341F-806R; 16S V3, 528F-706R) with a barcode. All PCRs were carried out with 15 μL of Phusion high-fidelity PCR master mix (New England Biolabs), 0.2 μM forward and reverse primers, and about 10 ng template DNA. Thermal cycling consisted of initial denaturation at 98°C for 1 min, followed by 30 cycles of denaturation at 98°C for 10 s, annealing at 50°C for 30 s, and elongation at 72°C for 30 s.

### PCR product quantification and qualification.

The same volume of 1× loading buffer (containing SYBR green) was mixed with PCR products, and electrophoresis on a 2% agarose gel was carried out for detection. PCR products were mixed in equidensity ratios. Then, mixtures of PCR products were purified with the Qiagen gel extraction kit (Qiagen, Germany).

### Library preparation and sequencing.

Sequencing libraries were generated using the TruSeq DNA PCR-free sample preparation kit (Illumina, USA) following the manufacturer’s recommendations, and index codes were added. The library quality was assessed on the Qubit 2.0 fluorometer (Thermo Scientific) and Agilent Bioanalyzer 2100 system. At last, the library was sequenced on an Illumina NovaSeq platform, and 250-bp paired-end reads were generated.

### Data analysis.

Sequence analysis was performed by Uparse software (Uparse v7.0.1001, http://drive5.com/uparse/) (43). Sequences with ≥97% similarity were assigned to the same operational taxonomic units (OTUs). A representative sequence for each OTU was screened for further annotation. For each representative sequence, the Silva138.1 database was used based on the Mothur algorithm to annotate taxonomic information. In order to study phylogenetic relationships of different OTUs, and the difference of the dominant species in different samples (groups), multiple sequence alignment was conducted using the MUSCLE software (version 3.8.31, http://www.drive5.com/muscle/) (44). OTU abundance information was normalized using a standard of sequence number corresponding to the sample with the fewest sequences. Subsequent analyses of alpha diversity and beta diversity were all performed based on these output-normalized data.

Alpha diversity was applied in analyzing complexity of species diversity for a sample through 6 indices, including Observed-species, Chao1, Shannon, Simpson, and ACE. All indices in our samples were calculated with Qiime2 (version Qiime2-202006) and displayed with R software (version 3.1.0). Beta diversity analysis was used to evaluate differences of samples in species complexity. Principal-coordinate analysis (PCoA) was performed to get principal coordinates from and visualize complex, multidimensional data. A distance matrix of weighted or unweighted UniFrac among samples obtained before was transformed to a new set of orthogonal axes, by which the maximum variation factor is demonstrated by the first principal coordinate, the second maximum one by the second principal coordinate, and so on. PCoA was displayed by the WGCNA package, stat packages, and ggplot2 package in R software (version 3.5.3). Unweighted pair-group method with arithmetic means (UPGMA) clustering was performed as a type of hierarchical clustering method to interpret the distance matrix using average linkage and was conducted with Qiime2 (version Qiime2-202006). LEfSe analysis was performed using LEfSe software (version 1.0), and the default setting of the linear discriminant analysis (LDA) score was 4. Metastats analysis uses R software (version 3.5.3) to perform permutation tests between groups under each classification level (phylum, class, order, family, genus, and species) to obtain the *P* value and then uses the Benjamini and Hochberg false-discovery rate method to correct the *P* value. The *q* value is obtained. Analysis of similarity (ANOSIM), MRPP, and Adonis analyses use the anosim, mrpp, and adonis functions of the R vegan package, respectively. Analysis of molecular variance (AMOVA) was performed using the mothur software amova function. Species with significant differences between groups were analyzed using R software (version 3.5.3) to do a between-group *t* test and plotted. Tax4Fun functional prediction was achieved by the nearest neighbor method based on the minimum 16S rRNA sequence similarity by extracting the KEGG database prokaryotic whole-genome 16S rRNA gene sequence and aligning it with the Silva SSU Ref NR database using the BLASTN algorithm (BLAST Bitscore >1,500) to establish a correlation matrix and to map the prokaryotic whole-genome functional information of the KEGG database annotated by UProC and PAUDA to the Silva database to implement the Silva database function annotation. The sequenced samples were clustered out of the OTU using the Silva database sequence as a reference sequence to obtain functional annotation information.

### Data availability.

The obtained metagenomic profiles have been uploaded into the NCBI SRA database and are accessible via the accession number PRJNA730380.
