# Supplementary material for: The Lumenal Microbiota Varies Biogeographically in the Gastrointestinal Tract of Rhesus Macaques
Source: Microbiol Spectr. 2022 May 2;10(3):e00343-22. doi: 10.1128/spectrum.00343-22 (PMC9241614; doi:10.1128/spectrum.00343-22)
Supplement: SUPPLEMENTAL FILE 1 — Supplemental material. Download spectrum.00343-22-s001.pdf, PDF file, 0.2 MB [file spectrum.00343-22-s001.pdf]

Table S1 Animal information and sampling locations.

Animal	Group	Age (years)	Sex	Weight (Kg)
1#	DU, JE, IL, CE, CO, RE	13	male	7.1
2#	DU, JE, IL, CE, CO	15	male	8.5
3#	DU, JE, IL, CE, CO, RE	13	male	7.2
4#	DU, JE, IL, CE, CO, RE	12	male	7.7
5#	DU, JE, IL, CE, CO, RE	15	male	8.3
6#	DU, JE, IL, CE, CO, RE	13	male	8.2
7#	DU, JE, IL, CE, CO, RE	13	male	7.7
8#	IL, CE, CO	16	female	7.0
